# A new linear regression-like residual for survival analysis, with application to genome wide association studies of time-to-event data

**DOI:** 10.1371/journal.pone.0232300

**Published:** 2020-05-04

**Authors:** Veronica J. Vieland, Sang-Cheol Seok, William C. L. Stewart

**Affiliations:** 1 Battelle Center for Mathematical Medicine, Abigail Wexner Research Institute, Nationwide Children’s Hospital, Columbus, OH, United States of America; 2 Department of Pediatrics, The Ohio State University, Columbus, OH, United States of America; 3 Department of Statistics, The Ohio State University, Columbus, OH, United States of America; Jilin University, CHINA

## Abstract

In linear regression, a residual measures how far a subject's observation is from expectation; in survival analysis, a subject's Martingale or deviance residual is sometimes interpreted similarly. Here we consider ways in which a linear regression-like interpretation is not appropriate for Martingale and deviance residuals, and we develop a novel time-to-event residual which does have a linear regression-like interpretation. We illustrate the utility of this new residual via simulation of a time-to-event genome-wide association study, motivated by a real study seeking genetic modifiers of Duchenne Muscular Dystrophy. By virtue of its linear regression-like characteristics, our new residual may prove useful in other contexts as well.

## 1. Introduction

In this paper we develop a new form of survival analysis residual with linear regression-like (LRL) properties, in a sense to be made clear in what follows. The simplest way to motivate this work is by describing a target application: discovery of genes that modify the Duchenne muscular dystrophy (DMD) phenotype.

DMD is an X-linked recessive disorder affecting ≈ 1 in 5,000 live male births [1, 2]. DMD involves progressive muscle tissue loss with replacement by fat and fibrotic tissue, and is currently without a cure. Patients typically become reliant on wheelchairs by early to mid-adolescence, but some maintain ambulation substantially longer, and age at loss of ambulation (LOA) is an important clinical indicator of disease progression. A great deal is known about the gene (*DMD*) that causes DMD, including the fact that modifier genes influence the rate of disease progression in a DMD mouse model [3, 4]; evidence for modifiers exists in humans as well [5–8]. The discovery of modifier genes in humans has implications both for therapeutics and for the design of DMD clinical trials.

Using data from the United Dystrophinopathy Project, a multisite consortium [9–11], we are currently engaged in a search for modifier genes under a Genome-Wide Association Study (GWAS) design. The sample comprises ≈ 500 DMD patients, among whom ≈ 60% have an observed age at LOA. The remaining individuals are censored (still ambulant at last contact or lost to follow up prior to LOA). There is one binary covariate in the model: steroid use prior to LOA. All subjects have been genotyped on a microarray chip, yielding approximately 1.5 million single nucleotide polymorphisms (SNPs) for analysis.

A standard approach to analyzing these data would be to perform survival analysis, say, under the proportional hazards (CPH) model [12], including both steroid use and genotype as covariates, performing the regression separately for each SNP. However, there are several drawbacks to this approach. One immediate issue is that the DMD dataset contains related individuals (brothers, cousins, etc.). Because relatives will be genotypically correlated, we would need to drop all but one individual from each family to satisfy the modeling assumptions of CPH regression, which would be wasteful of data. (Although partial- or pseudo-likelihood methods could perhaps be used, see, e.g., [13]. Note too that there are also circumstances in which dropping relatives is not even an option, e.g., in genetic linkage analysis where individuals without relatives are uninformative.) But also, we are interested in applying an approach focused on measurement of the strength of statistical evidence (rather than hypothesis testing), based on a quasi-Bayesian methodology implemented in the computer software package Kelvin [14].

Kelvin has several advantages in the context of GWAS data. It seamlessly handles mixtures of unrelated individuals and pedigrees; it can accumulate evidence for or against association, while handling unknown parameters of the trait model in a robust way under only mild distributional assumptions (in particular, it does not require normality for a quantitative trait at the population level [15]); and it can detect genotypic effects on phenotypic variances as well as on means. Kelvin also supports Bayesian sequential updating (see Appendix A) to accumulate evidence across data subsets, for example, based on mutation classes within the *DMD* gene. Trait-marker association analysis in Kelvin produces a posterior probability of linkage disequilibrium (PPLD), setting a prior probability π = 0.0004 of LD between two random loci based in part on empirical calculations [16], and the PPLD does not require adjustment for multiple testing [17, 18]. For all these reasons, Kelvin is ideally tailored for our application.

However, Kelvin has no direct mechanism for handling survival data, and layering a survival model on top of Kelvin’s underlying pedigree-based likelihood would be prohibitively complex. (For related work see, e.g., [19] and [20]; but these approaches would entail foregoing some of Kelvin’s other features.) Our first thought for the DMD analysis, therefore, was to run CPH regression and to use the resultant *residuals* as the phenotypes for analysis in Kelvin. Indeed, as long as one can find a good model with which to compute covariate-adjusted residuals, then replacing data by regression residuals could be a general approach for downstream analysis whenever the target application cannot itself appropriately handle complications such as censoring. (See, e.g., [21] for a related approach.)

Crucial to this approach, however, is the requirement that the residuals remain scaled in terms of the original data. Our scientific hypothesis is that modifier genes influence age at LOA. Whatever manipulations we make to the phenotype along the way, the scale of the residuals must maintain a meaningful relationship to LOA. If the residuals were based on linear regression, they would clearly have a suitable interpretation as measures of *how extreme is an individual’s observed LOA relative to expectation*; this interpretation is simply a rescaling of the original units of time. But CPH regression would generate a Martingale residual (MR) or deviance residual (DR), and at the outset we were unclear on whether MR or DR would retain this LRL interpretation vis a vis the underlying age-at-event phenotype. In this paper we argue that MR and DR do *not* lend themselves to an LRL interpretation, but that we can modify DR to do so.

The paper is organized as follows. In (2) we review MR and DR and discuss ways in which they thwart an LRL interpretation. We introduce a new form of residual, which we call an ordinary time-to-event (OTE) residual, so called because it affords an ordinary LRL interpretation in the context of time-to-event data (TE), and we compare OTE with MR, DR and with an ordinary linear regression residual (OLRR). Then in (3) we assess the comparative behavior of MR, DR, OTE and OLRR when used in conjunction with the PPLD for analysis of TE-GWAS data, using simulations designed to mimic the DMD application.

## 2. Various residuals for time-to-event data and their interpretations

Section 2.1 reviews the definitions of MR and DR and their interpretation; 2.2 introduces the OTE residual, and also the form of OLRR we will use for comparisons; 2.3 discusses handling of a covariate; 2.4 then further illustrates key aspects of MR, DR and OTE compared with each other and with OLRR.

### 2.1 MR, DR and Their Interpretation

The MR for individual *x*_*i*_ at survival time *t*_*surv*_(*x*_*i*_) is defined as

$$\text{MR}\left(x_{i}\right)=\delta_{i}-\hat{\text{CHF}}\left\{t_{surv}(x_{i})\right\}$$ (1)

where *δ*_*i*_ = 1 if the individual is uncensored and *δ*_*i*_ = 0 if the individual is censored; and $\hat{\text{CHF}}\left\{t_{surv}(x_{i})\right\}$ is the estimated cumulative hazard function evaluated at min[*t*_*fail*_(*x*_*i*_), *t*_*cens*_(*x*_*i*_)], the individual’s failure time and censoring time, respectively. (Eq 1 assumes fixed, rather than time-dependent, covariates [22, 23], as we have in our DMD application. Technically MR is an estimate, but for notational convenience we omit the caret over expressions for residuals.) The range of MR is (-∞, 1], and asymptotically its expectation is 0. MR(*x*_*i*_) is sometimes said to represent the observed # of events minus the expected # of events for the *i*^*th*^ individual over (0, *t*_*i*_], suggesting an LRL interpretation. Its range, however, is markedly non-LRL.

DR is then defined as:

$$\text{DR}\left(x_{i}\right)=sign\left[\text{MR}\left\{t_{surv}\left(x_{i}\right)\right\}\right]\times \sqrt{-2\left(\text{MR}\left\{t_{surv}\left(x_{i}\right)\right\}+\delta_{i}\log\left[\delta_{i}-\text{MR}\left\{t_{surv}\left(x_{i}\right)\right\}\right]\right)}.$$ (2)

Eq 2 “corrects” for the fact that MR is bounded above by 1 but unbounded below. DR maintains the sign of MR, while expanding MR values close to one and contracting large negative MR values to achieve a more symmetric distribution [22]. DR thus behaves more like a residual from linear regression than does MR. We focus here on DR for this reason, but points raised in the remainder of this section apply equally to MR.

There are three reasons why DR should not be interpreted in an LRL manner. First, as previously noted, for a censored individual DR is based on $\hat{\text{CHF}}\left\{t_{cens}(x_{i})\right\}$. However, the information we have regarding the individual’s actual failure time is only that *t*_*fail*_(*x*_*i*_) > *t*_*cens*_(*x*_*i*_). If we are interested in evaluating $\hat{\text{CHF}}\left\{t_{surv}(x_{i})\right\}$ at a value that lends itself to an LRL interpretation in terms of underlying failure times (as opposed to a value chosen for its utility in Martingale theory), then evaluating the CHF at *t*_*cens*_(*x*_*i*_) seems an arbitrary and unsatisfactory choice. There is a difference here between the time-to-event scale we seek to preserve and the number-of-events framework of DR.

Second, DR assigns a different value to *δ*_*i*_ for uncensored and censored individuals. This makes sense if we define *δ*_*i*_ as the observed number of events at time of evaluation. One consequence of this assignment, however, is that two individuals, one censored and the other uncensored, with the same covariate status and the same survival time, are guaranteed to have a difference of 1 in their MR, with a corresponding difference in DR. Whether these two individuals should have different residuals is one question; whether the difference between their MR values should always be exactly equal to 1 is another. (See 2.4 below for a detailed illustration of this issue.) To maintain an LRL interpretation, we would like the residual to reflect only how extreme is the individual’s survival time relative to expectation, but DR’s dependence on *δ*_*i*_ confounds such an interpretation.

The third issue applies equally to uncensored and censored individuals. In linear regression, an individual with observation equal to expectation would be assigned a residual equal to 0. But DR = 0 does *not* correspond to the situation in which an individual’s observed survival time is equal to the expected survival time, even for uncensored individuals; we consider this point in greater detail in 2.2.

In aggregate these issues introduce scaling issues for DR (and MR) that undercut an LRL interpretation. And as we will show below, ignoring the non-LRL qualities of DR has implications for downstream analyses.

### 2.2 OTE and OLRR

OTE is obtained by making three modifications to DR, designed to remedy the three issues raised above. First, for censored individuals, we define the *predicted* failure time *t*_*pred*_(*x*_*i*_) as the median time to event conditional on *t*_*fail*_(*x*_*i*_) > *t*_*cens*_(*x*_*i*_), which can be calculated from the estimated survival function $\hat{S}\left(t\right)$; *t*_*pred*_(*x*_*i*_) represents the information afforded by censoring time regarding an individual’s (expected) failure time. (Our reason for using the median rather than the mean will become clear below.) In the context of OTE, we therefore define $t_{surv}^{*}\left(x_{i}\right)$ as *t*_*fail*_(*x*_*i*_) for uncensored individuals and *t*_*pred*_(*x*_*i*_) for censored individuals.

Second, we set *δ*_*i*_ = *δ* for all *i*. Heuristically, this reflects the new definition of $t_{surv}^{*}\left(x_{i}\right)$ for censored individuals; we are in effect treating everyone as having had one event (either observed or predicted) at the time of evaluation. More importantly, this choice removes scaling anomalies between uncensored and censored individuals, as we return to below.

Third, we set *δ* = −log(0.5) for all individuals (uncensored and censored). The rationale for this choice is that CHF($\tilde{t}$) = −log *S*($\tilde{t}$) = −log(0.5) ≈ 0.7, regardless of the shape of *S*(*t*), where $\tilde{t}$ is the median survival time. Thus an uncensored individual with survival time $\tilde{t}$ is assigned a positive DR, while a censored individual with survival time $\tilde{t}$ is assigned a negative DR. By setting *δ* = −log(0.5) we ensure OTE = 0 if and only if $t_{surv}^{*}\left(x_{i}\right)$ is the median survival time. This is also our justification for defining *t*_*pred*_(*x*_*i*_) in terms of the *median* survival time rather than the predicted *mean*. The latter depends on the shape of $\hat{S}\left(t\right)$, so that there would be no fixed LRL meaning for a residual = 0.

To summarize, OTE is calculated as follows: $\hat{S}\left(t\right)$ is estimated in the usual way based on *t*_*surv*_(*x*_*i*_), i.e., *t*_*fail*_(*x*_*i*_) for uncensored individuals and *t*_*cens*_(*x*_*i*_) for censored individuals. (For family data, only unrelated individuals would be used for this step.) $\hat{S}\left(t\right)$ is used to calculate the median survival time $\tilde{t}$, and, for each censored individual (including any relatives, in the case of family data), the predicted median survival time *t*_*pred*_(*x*_*i*_) given *t*_*fail*_(*x*_*i*_) > *t*_*cens*_(*x*_*i*_). $\hat{S}\left(t\right)$ is also used to obtain the corresponding $\hat{CHF}$(*t*). A modified MR (MMR) is then calculated for each individual (including any relatives) as MMR = −log(0.5) − $\hat{CHF}\left\{t_{surv}^{*}\left(x_{i}\right)\right\}$. Finally a DR-like transformation is applied to MMR, setting *δ* = −log(0.5):

$$\text{OTE}\left(x_{i}\right)=sign\left(MMR\left(t_{surv}^{*}\left(x_{i}\right)\right)\right)\times \sqrt{-2(MMR\left(t_{surv}^{*}\left(x_{i}\right)\right)-\delta \;\text{log}\;\delta +\delta \;\text{ln}\left[\delta -MMR\left\{t_{surv}^{*}\left(x_{i}\right)\right\}\right])}.$$ (3)

(See Appendix B for details.)

We note that for censored individuals with *t*_cens_(*x*_*i*_) ≈ 0, $t_{pred}(x_{i})\approx \tilde{t}$, which yields a residual ≈ 0; as *t*_cens_(*x*_*i*_) increases, the corresponding residual decreases. Thus, as with MR and DR, there is no such thing as a positive OTE residual for a censored individual. Of course this produces an asymmetry with respect to uncensored individuals, but this asymmetry is inherent in the available information.

For purposes of comparison, we also consider OLRR, which is computed on the original time scale as would be done in ordinary linear regression:

$$\text{OLRR}\left(x_{i}\right)=\tilde{t}-t_{surv}^{*}\left(x_{i}\right).$$ (4)

(We center OLRR on the median, rather than the mean, in order to maintain comparability with OTE. OLRR is in the form “expected—observed,” where ordinarily we would use “observed—expected.” This is again for comparability, since for DR and OTE positive values represent individuals with earlier survival times and negative values represent individuals with later survival times.) OLRR is naïve with respect to the shape of CHF: it represents residuals on the original time scale ignoring the cumulative hazard function, whereas OTE transforms time differences onto a hazard scale. OLRR is therefore not correct for time-to-event data. (OLRR makes partial use of $\hat{S}\left(t\right)$, but only insofar as $\hat{S}\left(t\right)$ is used to obtain *t*_*pred*_(*x*_*i*_) in the first place.) But because we seek a residual that is OLRR-like in interpretation, OLRR provides a useful point of reference.

### 2.3 Handling the covariate

We restrict our attention here to a single binary covariate *y*, in keeping with our intended DMD application. In a regression setting (e.g., under the CPH model), a separate CHF is estimated for each level of *y*, and for the *i*^*th*^ individual MR is calculated based on the $\hat{\text{CHF}}$ corresponding to that individual’s covariate status; i.e., the covariate “adjustment” is made at the level of estimation of the CHF. The same is true for linear regression, where the residual is calculated relative to a covariate-adjusted conditional mean. Accordingly, DR and OTE take the covariate into account by estimating separate survival curves based on *y*; similarly, OLRR subtracts $t_{surv}^{*}\left(x_{i}\right)$ from the *y*-specific median.

For our purposes we need to place the resulting residuals on the same scale across levels of *y*, in order to effectively “remove” the covariate effect prior to downstream analyses. Therefore, before combining residuals across levels of *y*, they are standardized separately for each level of *y*, using $\frac{RES\left(x_{i}\right)-{\bar{RES}}_{y}}{s.d.\left(RES_{y}\right)}$, where ${\bar{\text{RES}}}_{y}$ is the mean of the residual distribution (RES = MR, DR, OTE or OLRR) for given *y*, and s.d.(RES_*y*_) is the corresponding standard deviation. (We note here that for OTE, this standardization is effectively a rescaling by *s*.*d*.(OTE_y_) without any additional shifting in the numerator, because ${\bar{\text{OTE}}}_{y}\approx 0$; see below.) All steps described in 2.2 above are carried out separately for each level of *y*, including calculation of $\tilde{t}$ and *t*_*pred*_(*x*_*i*_).

### 2.4 Additional contrasts among DR, OTE and OLRR

**Fig 1:**
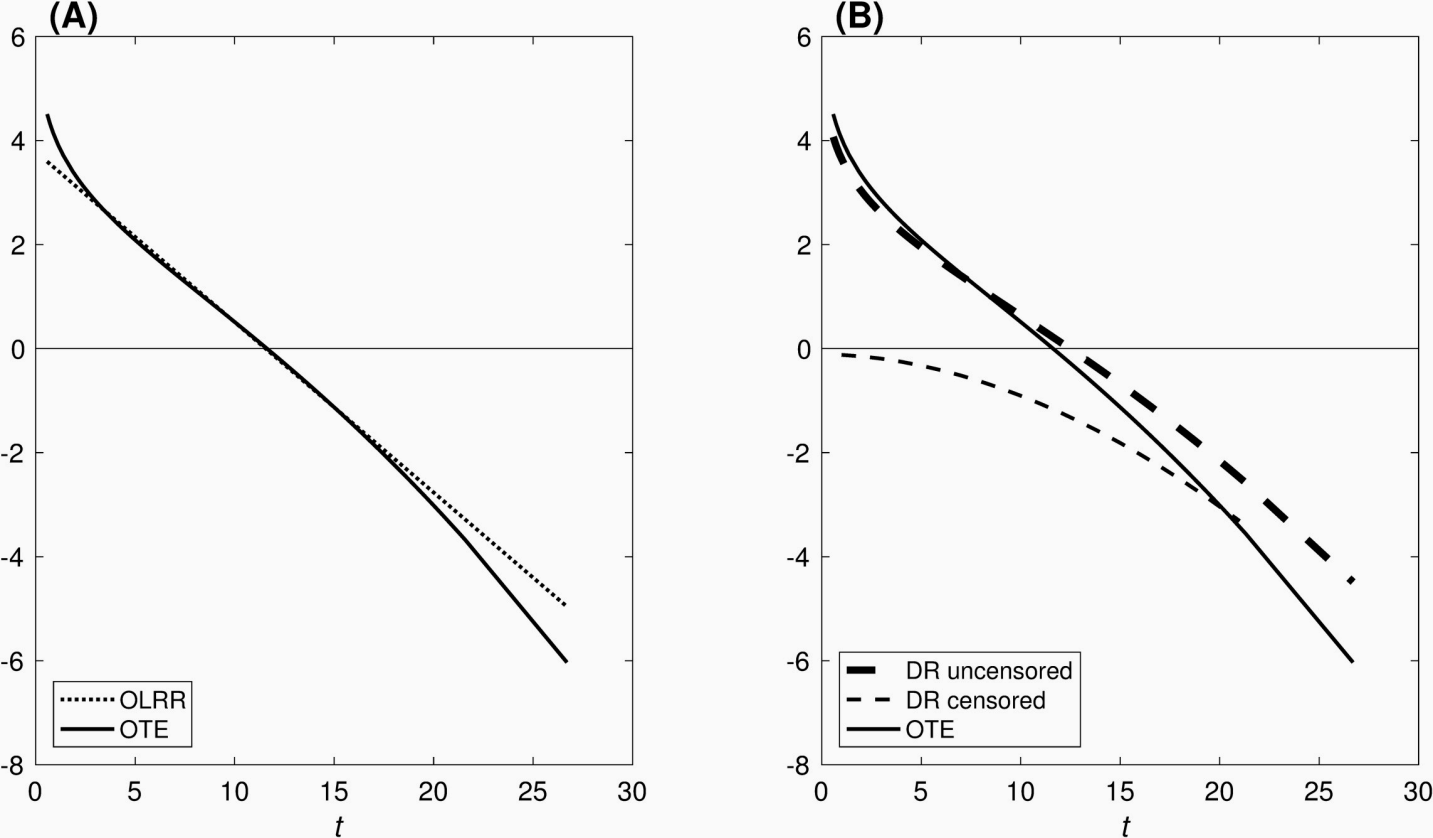
(A) OLRR and OTE, and (B) DR and OTE, as a function of time *t* at evaluation. Calculations shown here are based on a Weibull distribution with scale parameter *λ* = 12.89 and shape parameter *k* = 3.75, selected for illustrative purposes.

Our central claim is that the modifications to DR that lead to OTE change the *meaning* of the residual to a quantity that accurately reflects how extreme is an individual’s survival time relative to expectation (i.e., the median), on a hazard scale, after adjusting for covariate effects. Fig 1 illustrates this claim in part, by showing each residual as a function of time of evaluation. (For purposes of illustration, the calculations use a Weibull distribution without a covariate.) Recall that OTE and OLRR depend only on the (observed or predicted) survival time, without further distinguishing uncensored from censored individuals. Hence each is represented as a single line {Fig 1A}. We see that OTE preserves the same rank-ordering as OLRR. The differences between the two reflect the fact that, while OLRR is strictly linear in *t*, OTE correctly incorporates information from the hazard function. Note too that $\tilde{t}=11.69$ coincides with the intercept with *y* = 0 for both residuals. DR, however, not only differs in value from OTE (and OLRR) for given *t*, it also disrupts the rank-ordering across values for uncensored and censored individuals {Fig 1B}. Note too the slightly different placement of the intercept with *y* = 0, which for DR occurs at *t* = *λ* = 12.89.

**Fig 2:**
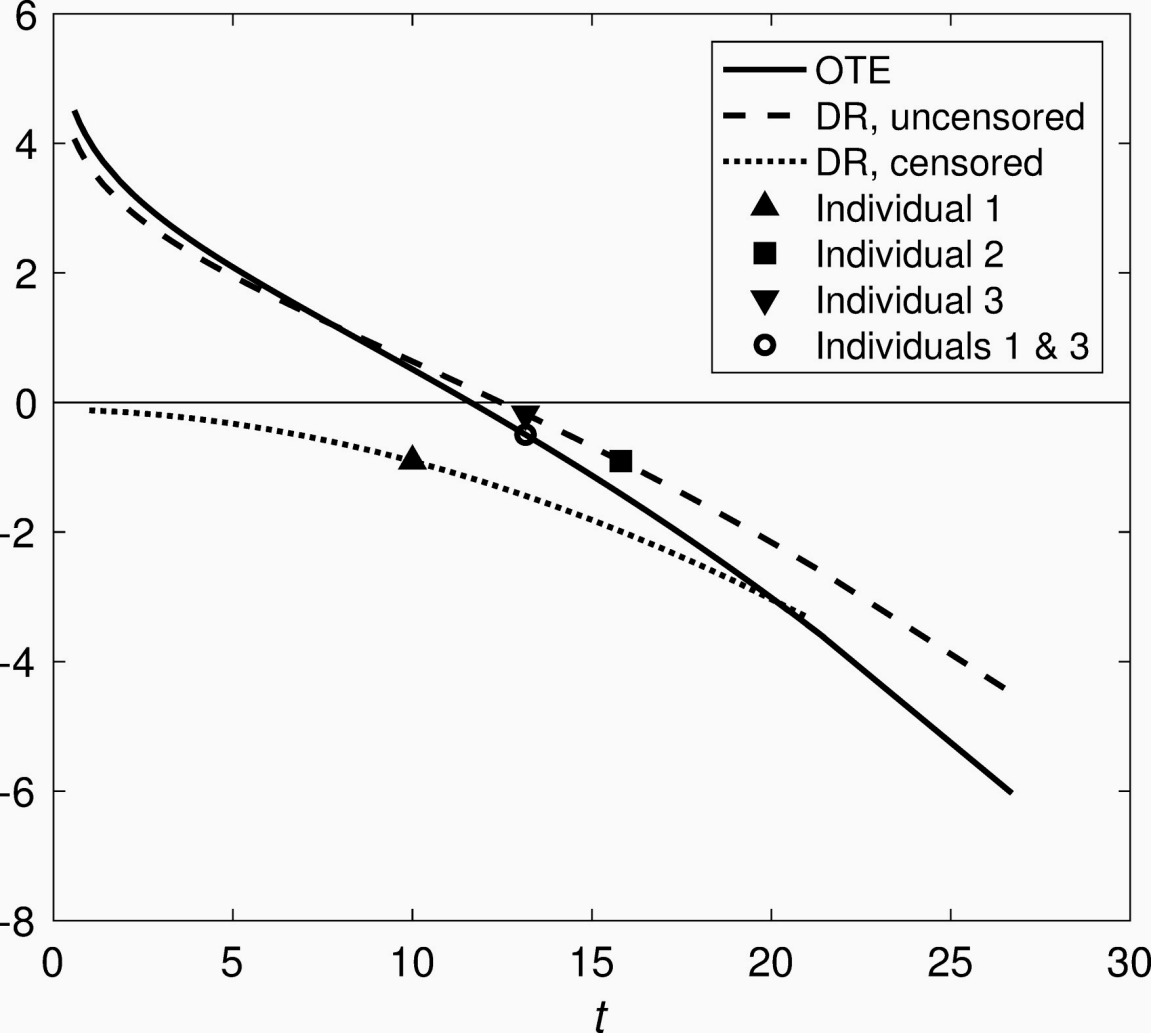
Illustration of key differences between DR and OTE. Calculations based on the same Weibull distribution used for Fig 1.

Fig 2 further illustrates this disruption of rank-ordering. For simplicity only DR and OTE are shown, since OLRR is quite similar to OTE. Consider 3 hypothetical individuals, as indicated on the figure. Individual 1 is censored at *t*_*cens*_(*x*_*1*_) = 10, yielding DR(*x*_*1*_) = −0.90. Individual 2 is uncensored, with failure time set such that DR(*x*_*2*_) = DR(*x*_*1*_), which gives *t*_*fail*_(*x*_*2*_) = 15.80. Individual 3 is uncensored with failure time equal to the value of *t*_*pred*_ corresponding to *t*_*cens*_ = 10, which for this particular CHF yields *t*_*fail*_(*x*_*3*_) = 13.15 and DR(*x*_*3*_) = −0.18. OTE assigns the same value to individuals 1 and 3, who share the same evaluation time (predicted or observed). DR, on the other hand, assigns different values to individuals 1 and 3, despite the fact that all the information we have regarding underlying failure times says that the two are equally extreme relative to the median. By contrast, DR assigns the same value to individuals 1 and 2, despite the fact that individual 1’s predicted failure time is less than individual 2’s observed failure time. Because of the other small differences between DR and OTE, OTE also assigns a different value to Individual 3 than does DR, with OTE(*T*_*3*_) = −0.50. Note that DR preserves the rank-ordering of MR; thus, while the residual values will differ between MR and DR, the differences illustrated above in rank-ordering and interpretation with respect to OTE remain for MR.

In summary: in contrast to MR or DR, OTE brings the scale for censored individuals into alignment with uncensored individuals, and situates the 0-point so that individuals with observation at the median are assigned OTE = 0. By replacing *t*_cens_(*x*_*i*_) with *t*_*pred*_(*x*_*i*_) for censored individuals, OTE more accurately represents the failure time information provided by censored individuals. Finally, OTE preserves the OLRR rank order with respect to deviation from the median, in (standardized) units of time-to-event, while incorporating information from $\hat{\text{CHF}}$(*t*). It is on this basis that we call OTE an LRL residual for time-to-event data. Table 1 summarizes these conclusions.

**Table 1:** Linear regression-like (LRL) properties for time-to-event residuals.

LRL Property of Residual	MR	DR	OLRR	OTE
When an individual’s event time is exactly at its expectation (i.e., the median) given covariates, the residual is 0	No	No	Yes	Yes
All individuals, whether censored or uncensored, are consistently rank-ordered with respect to available information regarding event time	No	No	Yes	Yes
Deviation of an individual’s event time from expectation is measured on the hazard scale	No	No	No	Yes

## 3. Simulation studies

In this section we evaluate the effectiveness of using OTE in the context of our intended genetic application using simulated data. Because our primary interest is in developing a residual for use in downstream Kelvin analyses, and because in reality the underlying genetic model cannot be known in advance of gene discovery, this is a situation in which simulated data can be far more informative than real data for purposes of methods validation. Section 3.1 describes the simulation methods, and 3.2 presents results.

### 3.1 Simulation methods

In order to mimic features of our DMD data set, our base model uses a sample size of N = 500 individuals, half of whom come from each of two covariate levels (*y* = 1, 2). Individuals were randomly assigned a genotype for a 2-allele locus (with alleles 1, 2) as a function of *q* = P(allele 1) = 0.5, assuming Hardy Weinberg Equilibrium. (This is in anticipation of genetic association analysis based on SNPs. Assuming *q* = 0.5 keeps things simple while generating sufficient data for all 3 genotypes; but it is also not unrealistic, since allele frequencies at modifier loci could be quite high in the general population.)

Failure time was simulated via a random draw from a mixture of normal distributions in the form *N*_*y = 1*,*k*_(*μ*_*k*_, *σ*_*k*_), for given genotype *k* = 11, 12, 22 and *y* = 1, and in the form *N*_*y = 2*,*k*_ (*μ*_*k*_ + 3, *σ*_*k*_) for *y* = 2. We also considered one model with a more complicated covariate effect (see below). The generating parameter values were chosen to mimic LOA in the real DMD data set for uncensored individuals with no history of steroid use ($\bar{\text{x}}=11.6$, *s*. *d*. = 3.4). All normal distributions were left-truncated at 0 in order to preclude non-positive age at event. (While the normal distribution was chosen for convenience, the resulting distribution was effectively Weibull; see SI.)

The genetic model was varied as shown in Table 2. Model 1 has no genotypic effect, so that the generating model is a single (non-mixed) normal distribution. Model 2 creates a simple additive mixture model for age-at-event while maintaining comparable $\bar{\text{x}}$ and *s*. *d*. at the population level. The remaining models vary effect size by increasing the genotypic variances (Model 3), introducing dominance (Models 4, 5), and by generating genotypic effects on variances as well as means (Model 6) or solely on variances (Model 7). Model 8 complicates the covariate effect. The models were chosen to illustrate a range of possible trait distributions, and are by no means intended to exhaustively cover what we might find in a real application.

**Table 2:** Simulation generating Models^†^.

Model	*μ*_11_ (σ_11_)	*μ*_12_ (σ_12_)	*μ*_22_ (σ_22_)
**1**	0 (1)	0 (1)	0 (1)
**2**	–0.5 (1)	0 (1)	0.5 (1)
**3**	–0.5 (1.25)	0 (1.25)	0.5 (1.25)
**4**	–0.5 (1.25)	0.5 (1.25)	0.5 (1.25)
**5**	–0.5 (1.25)	–0.5 (1.25)	0.5 (1.25)
**6**	–0.5 (0.5)	0 (1)	0.5 (1.5)
**7**	0 (0.5)	0 (1)	0 (1.5)
**8**	–0.5, 1.26 (1, 2)	0, 1.76 (1, 2)	0.5, 2.26 (1, 2)

^†^ Models shown on the standard normal scale for the *y* = 1 group. For Models 1–6, 3 years are added to the *y* = 1 means for the *y* = 2 group, as described in the text. For Model 8, *y* = 2 affects the means differently for the 3 genotypic groups in addition to affecting the variance; a comma separates the generating means (s.d.s) for *y* = 1, *y* = 2, respectively.

For each generating model, an individual was simulated based on a random draw of *t*_*e*_(*x*_*i*_) from the corresponding age-at-event (AE) distribution and an independent random draw of *t*_*o*_(*x*_*i*_) from an age-at-observation (AO) distribution. If *t*_*e*_(*x*_*i*_) < *t*_*o*_(*x*_*i*_), the individual was considered uncensored with failure time *t*_*fail*_(*x*_*i*_) = *t*_*e*_(*x*_*i*_); otherwise, the individual was considered censored with censoring time *t*_*cens*_(*x*_*i*_) = *t*_*o*_(*x*_*i*_). AO was simulated under a negative binomial distribution with *r* = 10, *p* = 0.4 in order to roughly mimic the censoring distribution in the real data, with ≈ 40% of individuals censored. We simulated only unrelated individuals.

For each simulated data set, we obtained $\hat{S}$_y_(*t*) and $\hat{\text{CHF}}$_y_(*t*), as a function of age *t*, via maximum likelihood estimation of a pair of 2-parameter Weibull distributions, one for each level of *y*, based on *t*_*fail*_(*x*_*i*_) for uncensored individuals and *t*_*cens*_(*x*_*i*_) for censored individuals. We verified visually that the Weibull distribution provided a reasonable fit to the simulated data in comparison to Kaplan Meier curves. We simulated 1,000 replicates per model for Models 2–8; for the “null” Model 1, we simulated 1,000,000 replicates. PPLD-RES (RES = MR, DR, OTE or OLRR) was calculated for each replicate, using the residuals as data, and distributions of the PPLD across replicates were obtained for each model.

Recall that the PPLD does not require normality or even symmetry of the trait distribution. Therefore the distribution of the residuals is not our primary interest here. However, additional information on these distributions is given in Appendix C.

### 3.2 Simulation results

Table 3 shows summary statistics of the PPLD distributions for the 8 models shown in Table 2. (Recall that PPLD is on the probability scale, with values < 0.0004 indicating evidence against association and values > 0.0004 indicating (some degree of) evidence in favor of association.) For the “null” (non-genetic) Model 1, we see that all four forms of residual return very small PPLDs on average, below the prior of 0.0004, indicating evidence against association, with very little variability across replicates. For the remaining models, and based on Table 3 alone, we might conclude that PPLD-OTE and PPLD-OLRR are virtually identical in their behavior; with PPLD-DR also working reasonably well for the models considered here, although with a lower mean in general, and even worse performance for MR. Recall too that MR and DR preserve rank-order with respect to one another, so that performance differences are due to scaling differences. This underscores the importance of having a residual that maintains appropriate scaling with respect to the scientific hypothesis, in our case, regarding time to event.

**Table 3:** Mean (standard deviation) of PPLD distributions for each Model in Table 1.

Model	PPLD-MR	PPLD-DR	PPLD-OTE	PPLD-OLRR
**1**	0.0002 (0.0002)	0.0002 (0.0002)	0.0002 (0.0002)	0.0002 (0.0002)
**2**	0.72 (0.41)	0.78 (0.37)	0.84 (0.33)	0.84 (0.32)
**3**	0.27 (0.39)	0.36 (0.42)	0.42 (0.44)	0.41 (0.44)
**4**	0.73 (0.41)	0.82 (0.33)	0.87 (0.28)	0.87 (0.28)
**5**	0.69 (0.42)	0.75 (0.37)	0.78 (0.36)	0.79 (0.35)
**6**	0.98 (0.12)	0.97 (0.12)	1.00 (0.01)	1.00 (0.01)
**7**	0.54 (0.43)	0.80 (0.34)	0.95 (0.17)	0.95 (0.16)
**8**	0.28 (0.39)	0.23 (0.36)	0.27 (0.39)	0.28 (0.39)

**Fig 3:**
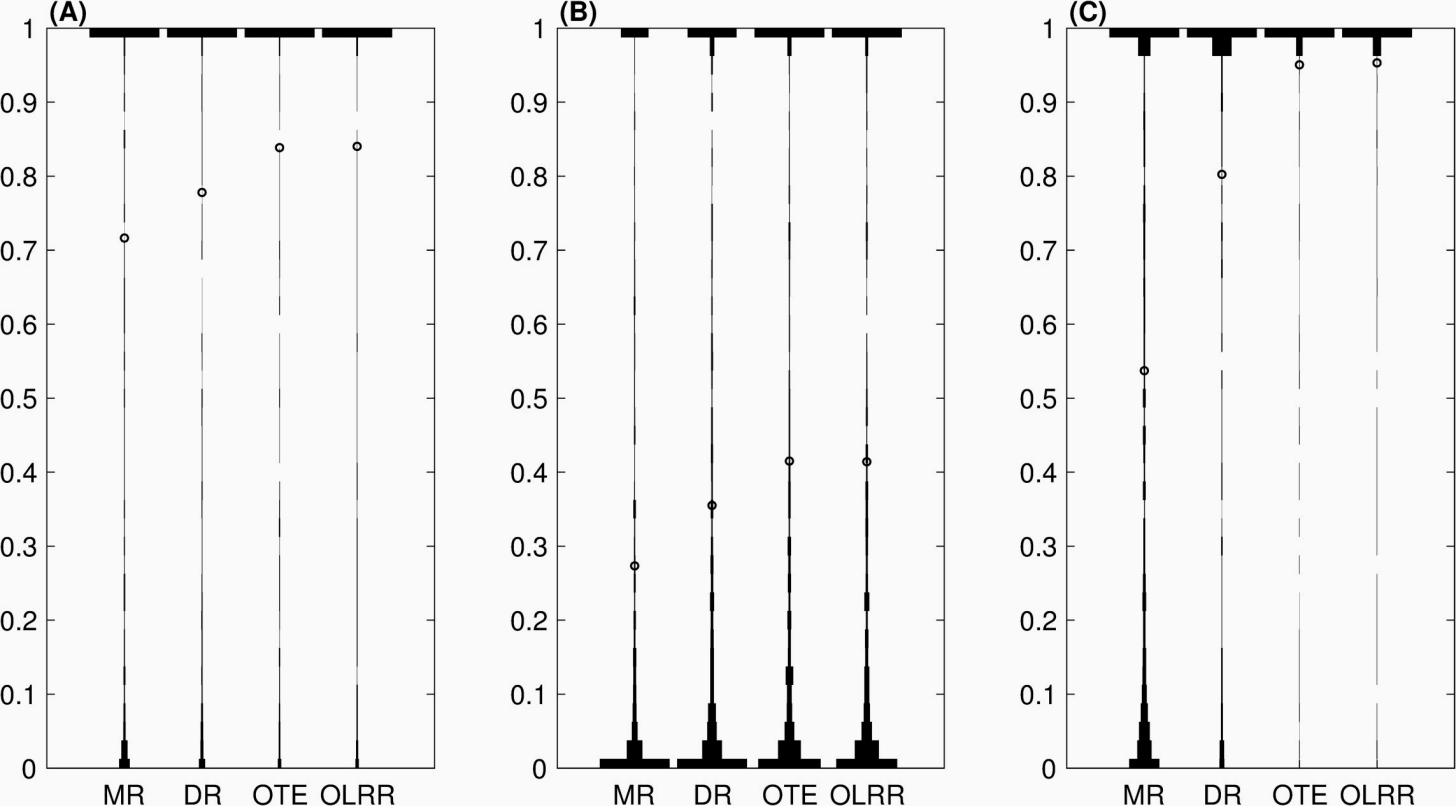
PPLD distributions (violin plots) for (a) Model 2, (b) Model 3 and (c) Model 7, using the indicated form of residual (MR, DR, OTE, OLRR) as input data. The mean of each distribution is marked with an ‘o’.

Fig 3 shows selected violin plots for the sampling distribution of each PPLD-RES, chosen to illustrate a model with high mean PPLD and similar standard deviations across PPLD-RES (Model 2); a model with lower means and similar standard deviations (Model 3); and a model with dissimilar standard deviations (Model 7). As can be seen, models for which the PPLD means are similar to one another can have different distributions depending on the form of residual, with the chances of obtaining a large PPLD in general higher for PPLD-OTE compared to PPLD-MR or PPLD-DR.

In addition, pairwise correlation coefficients between PPLD-MR or PPLD-DR and PPLD-OTE across replicates can be quite low, as shown in Table 4; even PPLD-OLRR and PPLD-OTE show lower correlations for some models. Thus in any given data set, depending upon the underlying genetic model (which is in practice always unknown), the PPLD can vary substantially depending on which form of residual has been used as data, even when the sampling distributions of the PPLD appear similar. This can impact rank-ordering of SNPs and lead to follow-up of different genes.

**Table 4:** Pair-wise correlation coefficients between PPLDs.

	Model							
	1	2	3	4	5	6	7	8
**MR & OTE**	0.21	0.77	0.75	0.69	0.83	0.22	0.33	0.83
**MR & OLRR**	0.19	0.76	0.75	0.68	0.83	0.18	0.32	0.85
**MR & DR**	0.26	0.86	0.83	0.75	0.90	0.70	0.58	0.87
**DR & OTE**	0.32	0.83	0.87	0.90	0.88	0.20	0.54	0.89
**DR & OLRR**	0.40	0.84	0.87	0.90	0.88	0.15	0.56	0.89
**OTE & OLRR**	0.82	0.99	0.99	0.99	0.99	0.75	0.96	0.98

## 4. Discussion

We have developed a novel OTE residual for use in conjunction with time-to-event data, and we have argued that OTE has an interpretation much like that of an ordinary linear regression residual. OTE was designed to satisfy certain requirements of scaling and interpretation. It is notable, then, that in operational terms (Table 3 and Fig 3) it out-performs the alternatives considered here in application to simulated TE-GWAS data. In general, a statistical method that retains more information and/or uses that information more accurately ought to, on average, outperform methods that rely on assumptions violated by the data or that distort the scaling of the data. Thus the superior operational characteristics of OTE, over a range of genetic generating models and across a large number of replicates, is indirect confirmation that OTE does indeed do what we intended it to do.

When designing and evaluating test statistics, operational behavior is important. But in measuring the strength of evidence for or against an hypothesis in any given data set, what counts is not just operational effectiveness, but more importantly, how accurately and efficiently the statistical method utilizes the relevant information in the data at hand. In our actual study of DMD, we have at present only the one data set to work with, and we need to know that our chosen outcome measure represents the evidence as accurately as possible in this particular data set. Key to this objective is ensuring meaningfulness for the measurement scale on which data are represented. The strikingly low correlations (Table 4) for some generating models between PPLD-MR or PPLD-DR and PPLD-OTE underscores this issue. If we are to take results in any one data set at face value, we must ensure up front that the form of residual we use accurately and reliably captures the information we set out to model: *how extreme is an individual’s survival time relative to expectation*. On this score we hope to have made a convincing case in favor of OTE.

While we have focused on application to TE-GWAS data, OTE could be useful in other contexts as well. In particular, in any setting for which it is desirable to remove covariate effects prior to combining data across covariate levels, these new residuals represent a methodology for so doing that retains a direct connection to the original scale of the dependent variable, simply shifting from survival time to the deviation of survival time from expectation, on a hazard scale, and expressed for each covariate level in units of level-specific standard deviations.

But of course, nothing we have presented guarantees that OTE is optimal in all settings. We have not, for example, considered the handling of more complex covariate structures, including continuous covariates or covariate interactions. OTE will also depend upon the choice of a functional form for the estimated survival function. While the Weibull distribution fit our simulated data well in comparison with Kaplan Meier curves, the robustness of downstream analyses to the fit of the survival curve would need to be considered in cases where that fit could be poor, and other survival models might need to be considered.

One additional aspect of our simulation results worth noting concerns the importance of including censored individuals, who are sometimes said to contribute relatively little to the power of survival analyses. In our application censored individuals turn out to contribute quite a bit. For instance, under Model 2, if we drop censored individuals prior to analysis the average PPLD-OTE drops from 84% to 12.5%. This is not particularly surprising given that censored individuals represent ≈ 40% of each data set, so that dropping them results in a substantial reduction in sample size. But it illustrates the importance of being able to properly incorporate censored individuals into such analyses. Recall too that the only information we have used for these individuals in constructing OTE is that *t*_*fail*_(*x*_*i*_) > *t*_*cens*_(*x*_*i*_). Even so, they prove to be quite informative; and of course, they are critical to proper estimation of ${\hat{S}}_{y}$ in the first place.

Finally, we note a difference in the handling of the covariate between what we are doing and the standard GWAS approach. The latter in effect imposes a random-effects model for the covariate, because the regression is performed separately at each SNP, with the covariate effect re-estimated each time. Along with the myriad other sources of sampling variability in the data, if a fixed effects model is more appropriate, then this practice introduces additional noise. By contrast, we have in essence assumed a fixed-effects model, factoring in the effect of the covariate just once in calculating the residuals. One disadvantage of this is that we might be sacrificing power to find genotype x covariate interactions. For instance, in our DMD data set, it is unclear whether using OTE as described here would support discovery of genes that influence response to steroids among DMD patients. This is an interesting topic for further investigation.

## Appendix A

### Brief description of the PPLD

Here we give a brief overview of the form of PPLD used in the main text; for additional details see [14]. The PPLD is based on the Bayes ratio (BR), defined as

BR=∫LR(γ)f(γ)dγ

where LR is a likelihood ratio representing “trait-marker association” in the numerator and “no association” in the denominator [24], and the single integral stands in for multiple integration over the vector ***γ*** = *μ*_11_, *μ*_12_, *μ*_22_, *σ*_11_, *σ*_12_, *σ*_22_, the means and standard deviations of three *t*-distributions, one for each of the three SNP genotypes [15]. For present purposes additional parameters of the likelihood are fixed as follows: recombination fraction *θ* = 0; standardized linkage disequilibrium (LD) parameter *D’* = 1 (see [24]); admixture parameter *α* = 1 (see [25]); disease minor allele frequency (MAF) = SNP MAF. These simplifications allow us to model genotypic effects of the SNP itself (whether direct effects or indirect through LD) on either ***μ*** or ***σ*** or both; without them one would need to include the MAF, which determines the mixing proportions for the genotypes, as an additional free parameter, which would result in a severely underdetermined parameter space and unreliable behavior for the PPLD.

BR is proportional to a likelihood for the marker data conditioned on the trait data, and for reasons having to do with ascertainment corrections [14, 26] it is integrated as a unit, rather than separately in the numerator and denominator like a Bayes factor [27], using highly accurate numerical methods [28]. Sequential updating across data subsets can be done by multiplying the BRs [13]. The underlying likelihood is based on the Elston-Stewart pedigree peeling algorithm ([29]. Let *π* be the probability that a randomly selected SNP is within detectable LD distance of a trait locus. Then the Posterior Probability of LD is simply $PPLD=\frac{\pi BR}{\pi BR+\left(1-\pi \right)}$.

## Appendix B

### Derivation of Eq 3

Define a function *g*(*x*) with parameter *b*:

g(x)=b−x−blog(b)+blog(x) (5)

This function has a maximum value of 0, which occurs at *x* = *b*. Let *x* = CHF(*t*). If we set δ = *b* = 1, the term *b log*(*b*) = 0 and we can write

g{CHF(t)}=1−CHF(t)+log{CHF(t)}. (6)

The usual formula for DR (Eq 2) can then be written by substituting Eq 6 under the square root sign, yielding

$$\text{DR}\left(t\right)=sign\left\{1-CHF\left(t\right)\right\}\times \sqrt{-2g\left\{CHF\left(t\right)\right\}}.$$ (7)

However, if we wish to have a function that maximizes at some other value of b≠1 then we need to restore the term *b log*(*b*). In particular, we would like to have OTE = 0 at *b* = − log(0.5). Setting *δ* = *b* = − log(0.5) ≈ 0.7, from Eq 5 we have

g{CHF(t)}=0.7−CHF(t)−(0.7)log(0.7)+(0.7)log{CHF(t)}. (8)

Substituting Eq 8 under the square root sign we arrive at the formula for OTE (Eq 3).

## Appendix C

### Distribution of residuals

**Fig 4:**
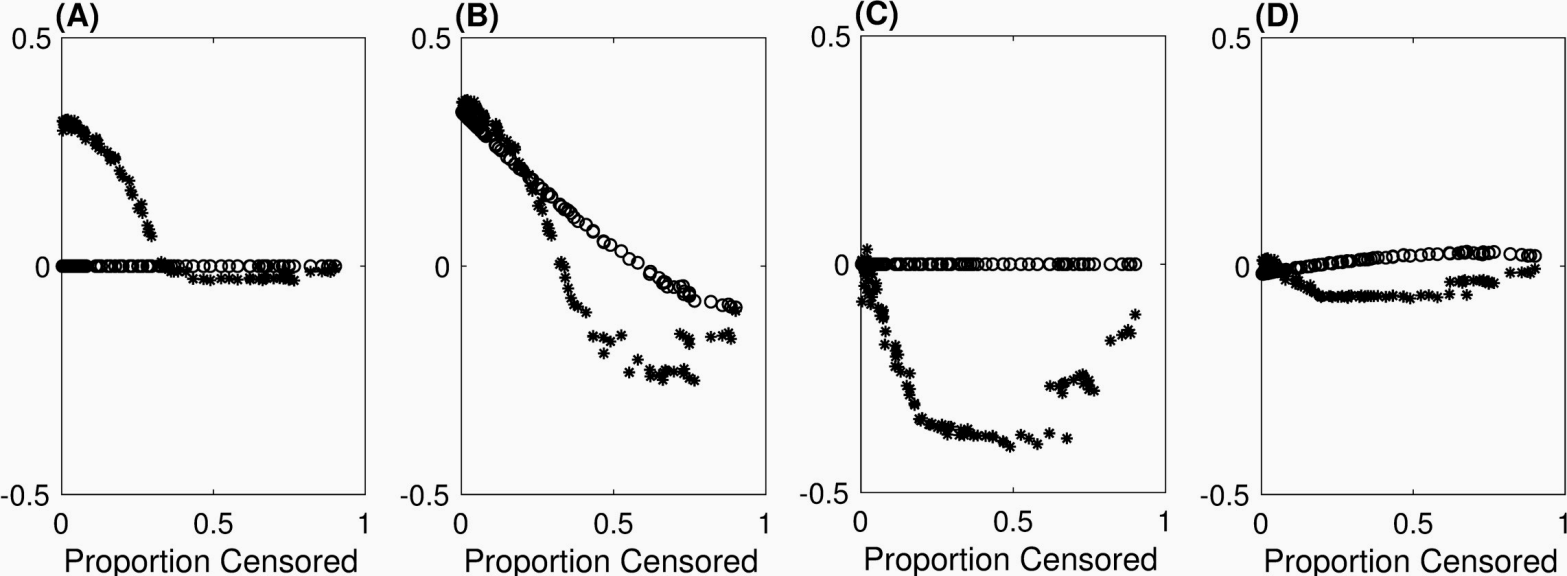
Simulated mean (o’s) and median (*’s) of RES distributions for (A) MR, (B) DR, (C) OLRR, (D) OTE.

Results shown here are based on 100 replicate data sets each of size N = 10,000, generated under Model 1 (*y* = 1). The age-at-observation (AO) distribution was varied by randomly sampling parameters of the negative binomial distribution from 2.5 ≤ *r* ≤ 14.5 and 0.25 ≤ *p* ≤ 0.35 in order to vary the proportion censored across replicates. Data were then generated and residuals calculated as described in the main text. (We confirmed that Weibull estimates across replicates varied from 12.70–12.93 (scale) and 3.61–3.92 (shape), producing virtually no visually discernable effect of the variable AO distribution on the survival function itself.) Fig 4 shows the mean ${\bar{z}}_{RES}$ and median ${\tilde{z}}_{RES}$ for each replicate, plotted as a function of the observed proportion of censored (OPC) individuals. As expected, ${\bar{z}}_{MR}=0$, and ${\bar{z}}_{OLRR}$ = 0 as well, regardless of OPC; ${\bar{z}}_{DR}$, however, depends on OPC, and for all three ${\tilde{z}}_{RES}$ depends on OPC. OTE is the closest to LRL behavior: ${\bar{z}}_{OTE}\approx 0$ and ${\tilde{z}}_{OTE}\approx 0$, regardless of OPC, implying that the underlying distributions of OTE residuals are highly symmetric.

## Supporting information

S1 Fig The figure below shows the fit of the Weibull distribution to the Kaplan-Meier curves for the simulated data under Model 1 (non-mixture model), based on all 1,000 replicates of N = 500 individuals each, for (A) y = 0 and (B) y = 1.
(TIF)
