# Supplementary figures and images for: A new linear regression-like residual for survival analysis, with application to genome wide association studies of time-to-event data
Source: PLoS One. 2020 May 4;15(5):e0232300. doi: 10.1371/journal.pone.0232300 (PMC7197860; doi:10.1371/journal.pone.0232300)

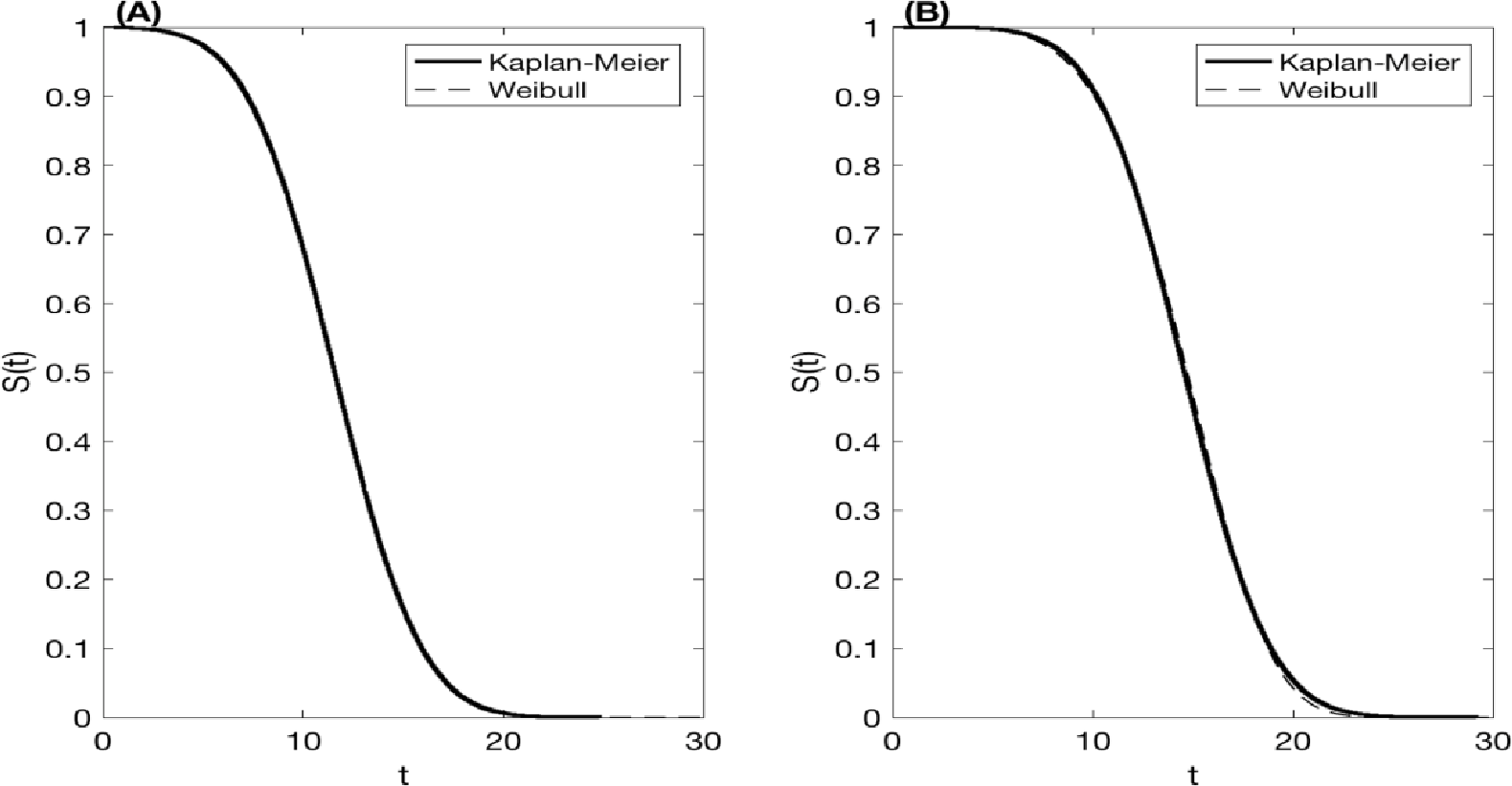

Supplement: S1 Fig — The figure below shows the fit of the Weibull distribution to the Kaplan-Meier curves for the simulated data under Model 1 (non-mixture model), based on all 1,000 replicates of N = 500 individuals each, for (A) y = 0 and (B) y = 1. (TIF) [file pone.0232300.s001.tif]
